# Community acceptance of services and effectiveness of health camps in high-risk areas of Karachi, Sindh, Pakistan, 2021

**DOI:** 10.3389/fpubh.2024.1498016

**Published:** 2025-01-08

**Authors:** Fayaz Hussain Abbasi, Jaishri Mehraj, Azeem Khowaja, Irshad Ali Sodhar, Shoukat Ali Chandio, Shumaila Rasool, Asif Ali Zardari, Imtiaz Hussain, Altaf Bosan, Erin M. Stuckey, Ahmed Ali Shaikh

**Affiliations:** ^1^Provincial Emergency Operation Center (PEOC), Government of Sindh, Karachi, Pakistan; ^2^Integral Global Health, Islamabad, Pakistan; ^3^United Nations International Children's Emergency Fund (UNICEF), Islamabad, Pakistan; ^4^National Stop Transmission of Polio (N-STOP) Program, Islamabad, Pakistan; ^5^World Health Organization, Islamabad, Pakistan; ^6^Center of Excellence in Women and Child Health, The Aga Khan University, Karachi, Pakistan; ^7^National Emergency Operation Center (NEOC), Islamabad, Pakistan; ^8^The Bill and Melinda Gates Foundation, Seattle, WA, United States; ^9^Riz Consulting, Islamabad, Pakistan

**Keywords:** health camps, childhood immunization, vaccination, polio eradication, mother and child health, Karachi, Pakistan

## Abstract

**Introduction:**

Health camps are organized to provide basic health services in underprivileged communities. This study was conducted to determine community acceptance and effectiveness of health camps in the high-risk areas for the polio program in Karachi, Pakistan.

**Methods:**

This cross-sectional survey was conducted at the health campsites in high-risk union councils (HRUCs) of four districts of Karachi, Sindh. The survey was carried out in three rounds after a polio vaccination campaign in June, August, and October 2021. In June and August, health camps were organized in eight HRUCs; in October, the scope was extended to 33 union councils. All health camps provided basic health services, maternal and child health services, and routine immunization.

**Results:**

In June 877, August 367, and October 383 respondents participated in the survey. The main services availed include consultation with a doctor (64% in June, 79% in August, 78% in October), followed by childhood vaccination (58% in June, 55% in August, 69% in October), and nutrition support (34% in June, 17% in August, 17% in October). Children’s immunization increased from 80% in June to 86% in August and 96% in October health camp rounds. Among parents who do not allow oral polio vaccine from polio teams at their doorstep, also vaccinated children at the health camps 48 (81%) in June, eight (80%) in August, and 13 (87%) in October.

**Conclusion:**

Health camps played a crucial role in building the reputation of the polio program among underserved communities in Karachi’s high-risk areas. Alongside routine immunization vaccination services, the provision of public health services such as permanent healthcare facilities, access to safe drinking water, proper waste disposal, and mother and child healthcare services, are crucial steps toward improving immunization and overall health outcomes and strengthening community trust.

## Background

Poliomyelitis is a highly infectious disease caused by wild poliovirus (WPV). It affects mostly children under 5 years of age and one out of 200 children infected with poliovirus is estimated to develop irreversible paralysis, with 5–10% of these cases resulting in death (1–3). Based on slight differences in the capsid proteins, the polioviruses are divided into three serotypes wild poliovirus type 1 (WPV1), type 2 (WPV2), and type 3 (WPV3). All three polioviruses are immunologically distinct (4). Type 2 was declared eradicated in September 2015 and the last case was detected in India in 1999. Type 3 poliovirus was declared eradicated in October 2019 and the last detected case occurred in Nigeria in 2012. The eradication of type 1 and type 2 polioviruses is a major public health success (4). Type 2 and type 3 caused less paralysis as compared to type 1. WPV1 is the most widespread and most commonly responsible for outbreaks and remains a target for complete eradication. Through vaccination efforts, WPV1 was greatly reduced globally, still, it is reported in a few countries mainly in Afghanistan and Pakistan (2). The oral polio vaccine (OPV) and inactivated polio vaccine (IPV) designed to protect against all three types of polioviruses, are playing crucial roles in global polio eradication efforts (1). Continuous surveillance and immunization are essential to keep polio eradication efforts on track.

There has been notable progress in reducing polio cases and their geographical spread globally (5, 6). Despite this, vaccine hesitancy to oral polio vaccine and routine immunization, poor access to maternal and child health care services, routine immunization services, water and sanitation services, malnutrition, and insecurity contribute to the remaining pockets of poliovirus circulation across high-risk areas of Pakistan. To enhance immunization coverage, there is a need for interventions that focus on community engagement and the provision of basic health services.

The Global Polio Eradication Initiative Technical Advisory Group for Pakistan and Afghanistan recommended in a meeting of February 2021 to organize well-coordinated health camps to provide health services to marginalized communities in Pakistan. Therefore, it was decided by the Emergency Operations Center’s (EOC) team to organize health camps during the sub-national immunization days (SNIDs) in June 2021 and subsequent supplementary immunization activities (SIAs) rounds to provide integrated health services delivery to the highest risk communities in Karachi, Sindh province (7).

The provincial government, Polio EOC Sindh, jointly conducted the health camps initiative and development partners, including Aga Khan University and Trust for Vaccines and Immunization (8). An agreement was reached to conduct portable camps during the polio vaccination campaign days in the identified union councils on a rotational basis at different places to amplify the reach and maximize community benefit. The health camps were organized in the areas based on the criteria of a high number of unvaccinated children being reported, a high number of chronic refusals, and repeatedly missed children being reported, and where access to health facilities was a challenge (9). Moreover, the survey was designed to gain insight into the number and type of beneficiaries (children, male, female), the type of services availed/preferred (vaccination, nutrition, consultation, medicines), and demand for other services (medical doctors, hospitals, vaccination centers, schools, safe drinking water, waste disposal, roads, etc.) as this information can be useful in planning impactful health camps in future rounds and enhance the reputation of the polio program in the community. The main purpose of the survey was to determine community acceptance and effectiveness of health camps in the high-risk union councils for the polio program in the Karachi division of Sindh province. The survey additionally aimed to identify community perceptions regarding hesitancy of vaccination through polio teams. Furthermore, the assessment was designed to inform the possible interventions to enhance acceptance of polio vaccination in underserved communities.

## Methods

This cross-sectional survey was conducted at the health campsites in high-risk union councils (HRUCs) and super high-risk union councils (SHRUCs) as designated by the Pakistan polio program in the Karachi division of Sindh province. Karachi Union Councils are categorized into Super High Risk, High Risk, Medium Risk, and Low-Risk categories based on Scocio-demographic, Immunological and Socio-economic profile. The Risk is multifold in Union Councils where the overall situation is poor. In Karachi, 39 Union Councils are recognized as HRUCs including 12 SHRUCs based on the criteria that; it is a polio reservoir where persistent poliovirus circulation is detected as either polio human cases or poliovirus in the sewage environmental sites is detected for more than 2 months, the population is underserved and dense with poor health structure, clustering of households refusing childhood vaccinations, or settlements of mobile/migratory populations. In addition, the immunization level is low for polio and other routine vaccines. HRUCs are normally the adjacent Union Councils to SHRUCs with poor campaign indicators such as a high percentage of missed children, and Union Councils that failed in lot quality assurance sampling due to operational gaps reported in previous multiple campaigns (10, 11). Those children who could not receive the OPV during an SIA as they were not present at home (not available) at the time when polio teams visited their house or their parents refused vaccination (refusals) are called missed children in the polio program of Pakistan.

**Figure 1:**
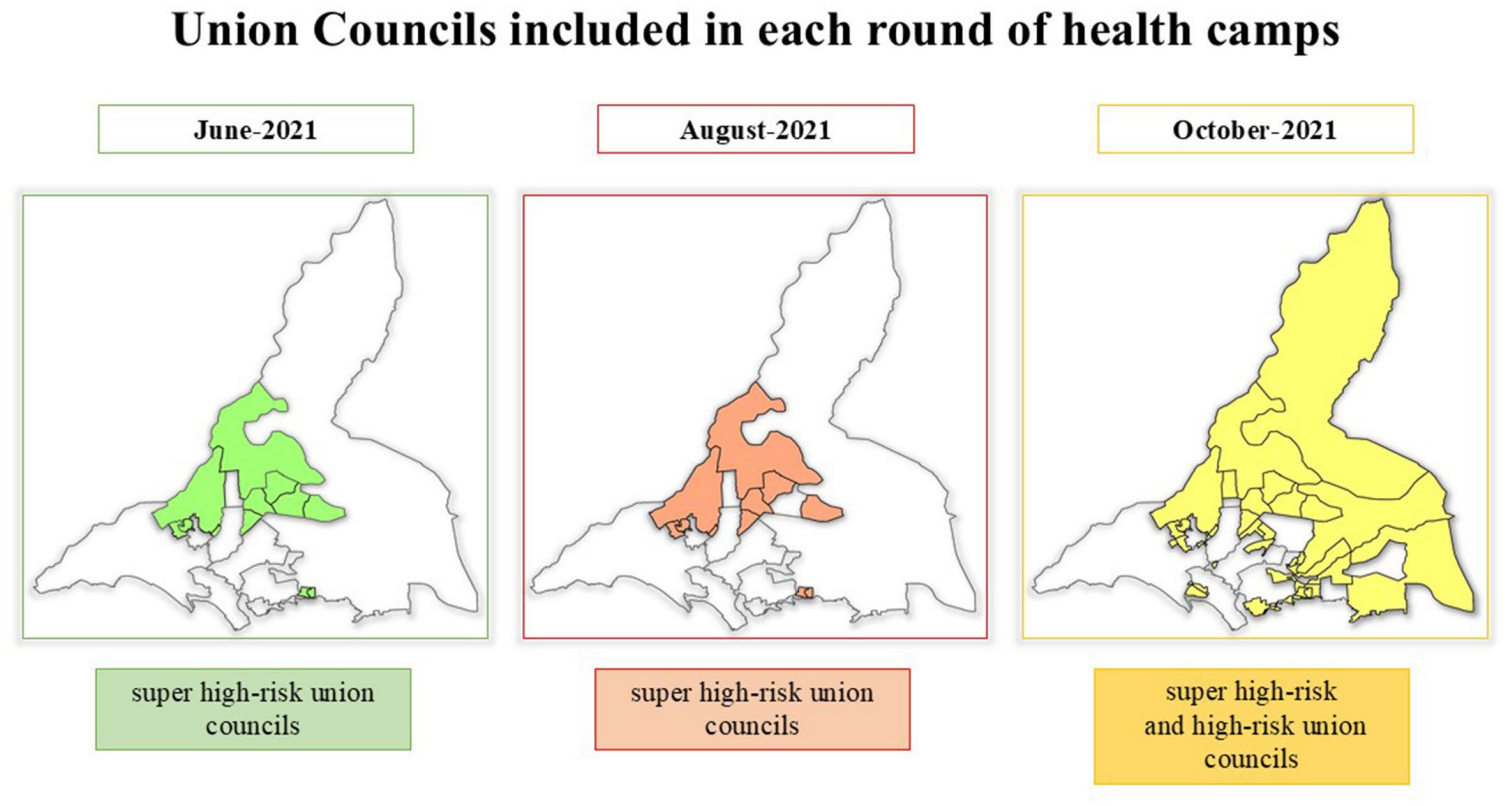
Union Councils included in each round of health camps in Karachi, Sindh, Pakistan.

In June and August 2021, health camps were organized in eight SHRUCs whereas in October 2021 the scope was extended to 33 union councils including 8 SHRUCs and 25 HRUCs. Figure 1 shows the Union Councils included in each round of health camps. The SHRUCs of Karachi divisions include Gujro (Gadap town, district East), Muzaffarabad and Muslimabad (Landhi town, district Malir), Islamia colony (SITE town, district West), Chishti Nagar (Orangi town, district West), Ittehad town (Baldia town, district West), Manghopir and Songal (Gadap town, district West). The areas or sites selected for health camps were based on the number of children missed for OPV vaccination during SIAs, the number of children missed for multiple rounds also referred to as persistently missed children (PMCs), and children recorded as having zero doses of OPV and who did not receive any injectable IPV from routine immunization. The site selection in the second and third rounds of health camps was irrespective of the selection of the same site in the previous rounds.

The health camp implementation teams collaborated with the polio program, which provided social mobilizers to support the health camps. These social mobilizers moved from house to provide caregivers with clear directions for the locations of health campsites and services offered. Mosque announcements, banner placements in catchment areas, and megaphone announcements were also part of social mobilization activities. District Health Officers provided furniture such as tents, chairs, and tables for the health camps, engaged philanthropists, and community-based organizations, arranged medicines, and provided logistic support. The select health camps that offered nutrition components included nutrition corners through an accelerated action plan program for nutrition. The district administration team and polio program partner staff provided support and monitored health camps.

A survey data collection tool was designed and administered to collect information on variables such as distance to health campsites, access and availability of different health services in nearby areas, sources of information about health camps, types of services provided, types of other services required, reasons for polio vaccine refusal, any issues in accessing the health services at nearby government health facilities or polio vaccination through teams at doorstep, number of children, ages of the participants, participant gender, and awareness about poliomyelitis (clinical outcomes, transmission, and prevention). The survey data collection tool is provided as a Supplementary material. A total of 12 surveyors were recruited to conduct the interviews with the beneficiaries at health camps across each of the selected Union Councils. Surveyors received training before implementing the survey. After participants availed the services of the health camps, and before they left the camp site the exit interviews were conducted by the surveyors.

The data was entered directly in an online Google form, extracted in Excel, and analyzed through SPSS v.21. Categorical data is presented in frequencies and percentages and a Pearson chi-square test is applied to evaluate the association between two variables, and *p*-value <0.05 was considered statistically significant association between the variables. The information was collected from those participants who came to avail of the services at the health camps sites. All study participants were adults and the data collection tool was not administered to any children. The study aims and objectives were explained to the participants and written informed consent was obtained before the start of the interview from each person who agreed to participate in this study. The names of the respondents were not disclosed to any person, or in any report or publication. Confidentiality was maintained throughout the study period. Ethical approval was obtained from the Provincial Bioethics Committee of the Director General Health Services, Government of Sindh.

## Results

The survey was conducted following three rounds of polio vaccination campaigns in Karachi, Sindh province, that took place in June, August, and October 2021.

In June 2021, the camps operated for 7 days starting from day one of the campaign from 9:00 AM to 3:00 PM daily during SNIDs June 2021 campaign days from 7th June to 13th June 2021. The target population for health camps was a total of 4,413 children reported as missed during the previous March 2021 polio campaign, including 2,310 refusals and 2,103 children that were not available. Moreover, 1,112 zero-dose (unvaccinated) children and 1,065 persistently missed children were also reported from the same areas. A total of 55 health camps (1 camp/union council/day) were implemented during June.

Services varied slightly at the health camps depending on the district. All 55 health camps provided basic health services, maternal and child health care services, and routine immunization. In addition, nutrition and birth registration services were also provided at the two health camps in Ittihad Town (district West) and Gujro (district East). The providers delivering services at health camps included male doctors, female doctors, Lady Health Workers (LHWs), vaccinators, dispensers, and social mobilizers. Steps were taken to ensure staff members, especially social mobilizers, had the same ethnic and linguistic profile as the community reached by the health camps.

In the August 2021 polio campaign round, 32 health camps were implemented over 2 days starting after the polio campaign concluded from 31st August to 1st September. The target was 3,024 missed children including 1,311 refusals and 1,713 children that were not available as reported during the August 2021 polio campaign. A total of 868 zero-dose children and 218 persistently missed children were also part of the target.

In October 2021, a total of 82 health camps operated over 4 days after the completion of the September NIDs campaign (from 5th to 8th October 2021). The target was 4,502 missed children, including 2,250 refusals and 2,252 that were not available during the preceding September 2021 NIDs. 3,765 zero-dose children and 1,365 persistently missed children were also included. Supplementary Figure 1 shows the Union Councils included in each round of health camps (Supplementary Figure 1).

In June 904 people approached, among them 877 (97.0%) agreed to participate in the study. In the August health camp round, a total of 379 beneficiaries approached and 367 (96.8%) agreed to participate in the study. In the October health camp round, a total of 396 beneficiaries of health camps were approached to get consent to participate in the study, and among them, 383 (96.7%) agreed to participate and were included in the study. No further information was collected from the people who were not willing to participate in the study.

In June, interviews were conducted with 877 respondents, in August 367, and in October 383 respondents. Across all three rounds, 56% of respondents were from the West Karachi district. Questions related to the accessibility of health camps were asked only in the June round and 418 (47.7%) respondents stated that camps were <100 m away from their homes and took 10–15 min to get there. Additionally, 394 (44.9%) reported a distance from 0 to 500 m, 58 (6.6%) reported a distance of 501–999 m, and only 7 (0.8%) reported a distance of 1,000 m or more. The time spent to arrive at the health campsite by walking from home was reported as 5 min or less by 381 (43.4%) respondents, 10–15 min by 418 (47.7%), 20–25 min by 60 (6.8%) respondents and >30 min by 18 (2.1%) survey respondents.

Female caregivers were the main beneficiaries of the camps, ranging from 698 (79.6%) in June to 300 (81.7%) in August, and 317 (82.8%) in the October round. The minimum age of respondents was 18 years in all rounds and the maximum age was 90 years, with a mean (standard deviation) of 32.5 (9.6) in June, 31.8 (11.1) in August, and 31.8 (8.4) in October 2021 rounds. A minimum of one to a maximum of seven household members of the survey participant had access to health camp services. 1,808 beneficiaries accessed services in June, 841 in August, and 733 in October. Polio vaccination teams were the main source of information reported among the health camp beneficiaries accounting for 723 (82.4%) respondents in June, 319 (86.9%) in August, and 316 (82.5%) in October 2021, followed in all three rounds by neighbors and mosque announcements. Most respondents attended the health camps with 2–4 other family members, and 768 (87.6%) of participants in June, 327 (89.1%) in August, and 344 (89.8%) in October came to the health camps accompanied by children. The main services received by the participants at the health camps included consultation with a doctor [544 (63.7%) in June, 291 (79.3%) in August, 297 (77.5%) in October], followed by childhood vaccination [492 (57.6%) in June, 201 (54.8%) in August, 266 (69.5%) in October], and nutrition support [288 (33.7%) in June, 62 (16.9%)% in August, 66 (17.2%) in October]. Availability of free Medicine was also the main response 311 (84.7%) in August, and 311 (81.2%) in October, when asked about services availed by the participants, but this option was not part of the survey data collection tool implemented in the June round. Table 1 shows the characteristics of study participants in each round of health camps.

**Table 1:** Characteristics of study participants in each round of health camps conducted in high-risk union councils of Karachi, Sindh, Pakistan.

Variables	June 2021	August 2021	October 2021
	(*N* = 877)	(*N* = 367)	(*N* = 383)
	%	%	%
Districts			
East	13.0	9.5	9.1
Keamari	8.0	9.3	24.3
Malir	21.3	14.2	11.0
West	57.7	67.0	55.6
Gender			
Female	79.6	81.7	82.8
Male	20.4	18.3	17.2
Age (mean SD)	32.5 (9.6)	31.8 (11.0)	31.8 (8.4)
^*^Source of information about health camps			
Polio team	82.4	86.9	82.5
Neighbors	18.0	19.9	23.5
Announcements (mosque, miking)	9.8	2.2	2.9
Participants with children			
No	12.5	10.9	10.2
Yes	87.5	89.1	89.8
^*^Services availed at this health camp			
Consultation with doctor	63.7	79.3	77.5
Medicines	–	84.7	81.2
Childhood vaccination	57.6	54.8	69.5
Nutrition support	33.7	16.9	17.2
Pregnant women vaccination	10.3	2.5	1.6
Family members of survey participants availed services of the health camp			
One	23	89	135
Two	257	149	170
Three	341	80	58
Four	178	36	16
Five	78	9	4
Six	0	3	0
Seven	0	1	0
Children received any vaccination at the health camp	(*N* = 547)	(*N* = 294)	(*N* = 321)
No	19.7	14.3	4.0
Yes	80.3	85.7	96.0
Satisfaction with the service providers’ (staff) behavior at the Health Camps	(*N* = 876)	(*N* = 367)	(*N* = 383)
Not satisfied	0.8	0.8	0.3
Partially satisfied	11.1	10.9	7.6
Satisfied	88.1	88.3	92.2
Camps are useful and should happen again.	(*N* = 876)	(*N* = 367)	(*N* = 383)
No	1.0	0	1.0
Yes	99.0	100	99.0

* Multiple response variables.

The proportion of missed children attending the health camps who received immunization services increased from 439 (80.3%) in June to 252 (85.7%) in August and 308 (96.0%) in October health camp rounds. Routine immunization utilization at the nearest health facility also increased from 647 (84.2%) in June to 289 (88.4%) in August and 318 (92.4%) in October as reported during the health camp rounds. More than 90% of respondents reported willingness to receive OPV vaccination from polio teams at their doorsteps [700 (91.1%) in June, 315 (96.3%) in August, and 326 (94.8%) in October] during SIAs. In all three rounds of health camps, many participants did not know what would happen if a child had a polio infection [136 (15.5%) in June, 99 (27.0%) in August, and 120 (31.3%) in October]. Similarly, significant proportions of participants were not aware of how poliovirus is transmitted [244 (27.9%) in June, 139 (37.9%) in August, and 118 (30.8%) in October], and did not know about preventing polio through the use of OPV or IPV vaccination [125 (14.3%) in June, 115 (32.4%) in August, and 120 (31.3%) in October]. Additional responses related to the knowledge, attitude, and practices of survey respondents regarding polio and routine immunization vaccines in each round of health camps conducted in high-risk union councils of the Karachi division of Sindh province are shown in Table 2.

**Table 2:** Knowledge, attitude, and practices of study participants regarding polio and routine immunization vaccines in each round of health camps conducted in high-risk union councils of Karachi, Sindh, Pakistan.

Variables	June 2021	August 2021	October 2021
	(*N* = 877)	(*N* = 367)	(*N* = 383)
	%	%	%
**Do you take your child to the nearest health facility for routine EPI Vaccination?**	(*N* = 767)	(*N* = 327)	(*N* = 344)
No	15.8	11.6	7.6
Yes	84.2	88.4	92.4
^**†**^ **EPI card retention**		(*N* = 289)	(*N* = 318)
No	–	37.0	34.0
Yes	–	63.0	66.0
^*^ **Why do you not prefer routine vaccination of your children?**	(*N* = 121)	(*N* = 38)	(*N* = 26)
Do not know about the nearest EPI	8.3	26.3	19.2
Facility	14.0	13.2	50.0
Not sure about vaccine quality	19.8	2.6	7.7
Afraid of side effects	30.6	23.7	3.8
Misconception	19.0	23.7	7.7
Prefer private vaccination	3.3	5.3	3.8
Do not give importance to vaccination	–	15.8	23.1
Other	39.7	23.7	11.5
**Willingness for polio vaccination through teams at doorstep**	(*N* = 768)	(*N* = 327)	(*N* = 344)
No	8.9	3.7	5.2
Yes	91.1	96.3	94.8
**Preferred site for OPV**	(*N* = 68)	(*N* = 12)	(*N* = 18)
From a private health facility	33.8	8.3	–
From a government health facility	4.4	8.3	30.0
From a local general practitioner	4.4	0	0
Other	13.2	0	0
Do not prefer polio vaccine	55.9	83.3	70.0
^*^ **Why do you not prefer the polio vaccination of your child?**	(*N* = 68)	(*N* = 12)	(*N* = 18)
Religious	19.1	25.0	44.4
Not sure about the content of the vaccine	48.5	58.3	44.4
Repeated visits	14.7	33.3	22.2
Private Doctor’s advice	30.9	0	5.6
The polio team’s behavior	0	0	0
Other	25.0	8.3	16.3
^*^ **Do you know, what happens if a child gets Polio?**			
Paralysis	79.9	68.7	62.4
Death	10.8	4.4	6.3
Do not know	15.5	27.0	31.3
^*^ **What do you think about how polio is transmitted in children?**			
Contaminated water	23.5	30.2	36.0
Contaminated food	33.8	21.8	25.3
Poor hygiene	37.8	34.1	36.0
Not vaccinated	24.7	25.1	31.1
Do not know	27.9	38.7	30.8
^*^ **How polio can be prevented?**			
Oral Polio vaccine (OPV)—oral drops	84.2	65.4	62.1
Inactivated Polio vaccine (IPV)—injection	25.9	21.3	27.7
Do not know	14.3	32.4	31.3

† EPI card retention was not part of the survey tool in the June round.

* Multiple response variables.

**Figure 2:**
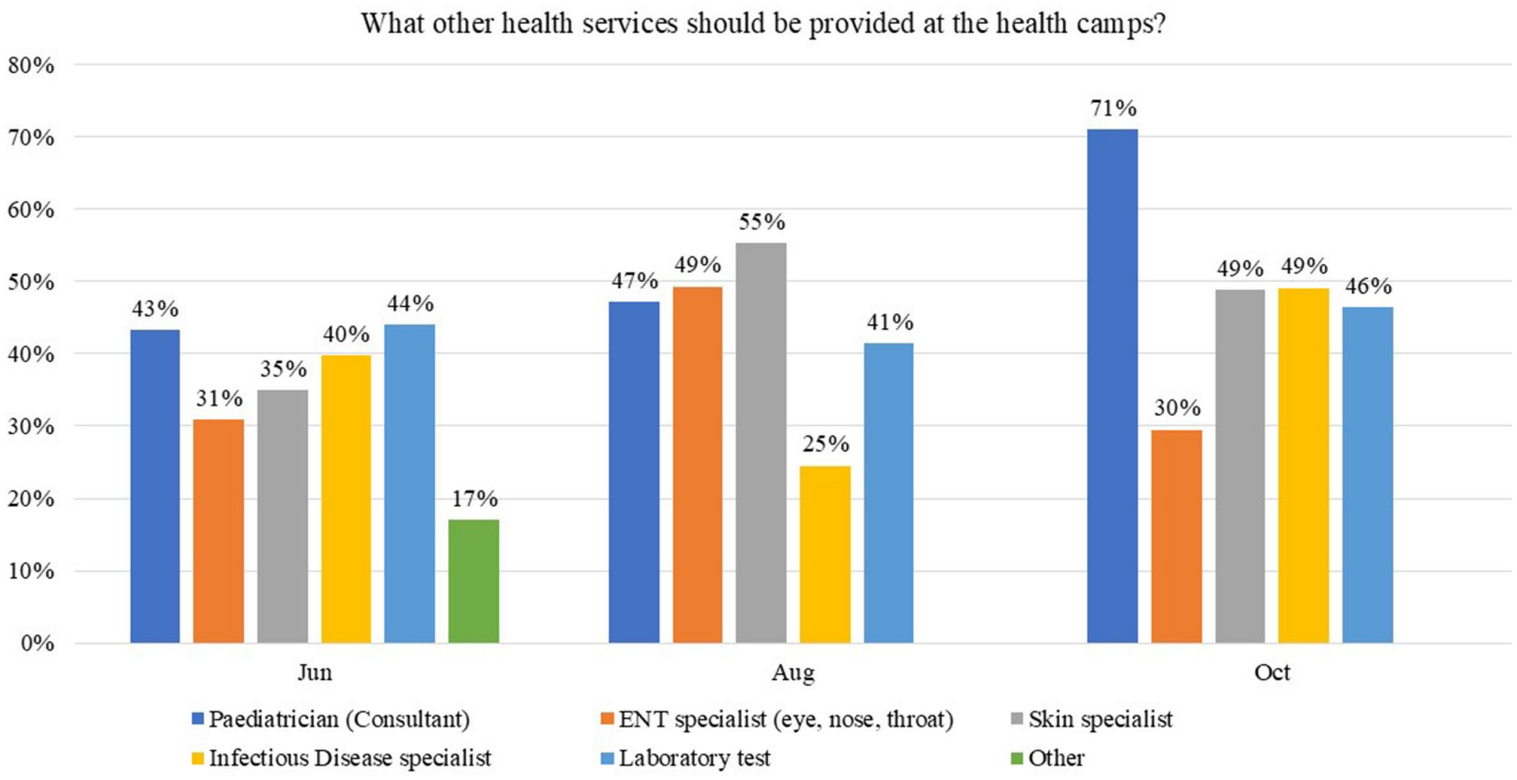
Participants’ responses on what other services should be provided at health camps in Karachi, Sindh, Pakistan.

**Figure 3:**
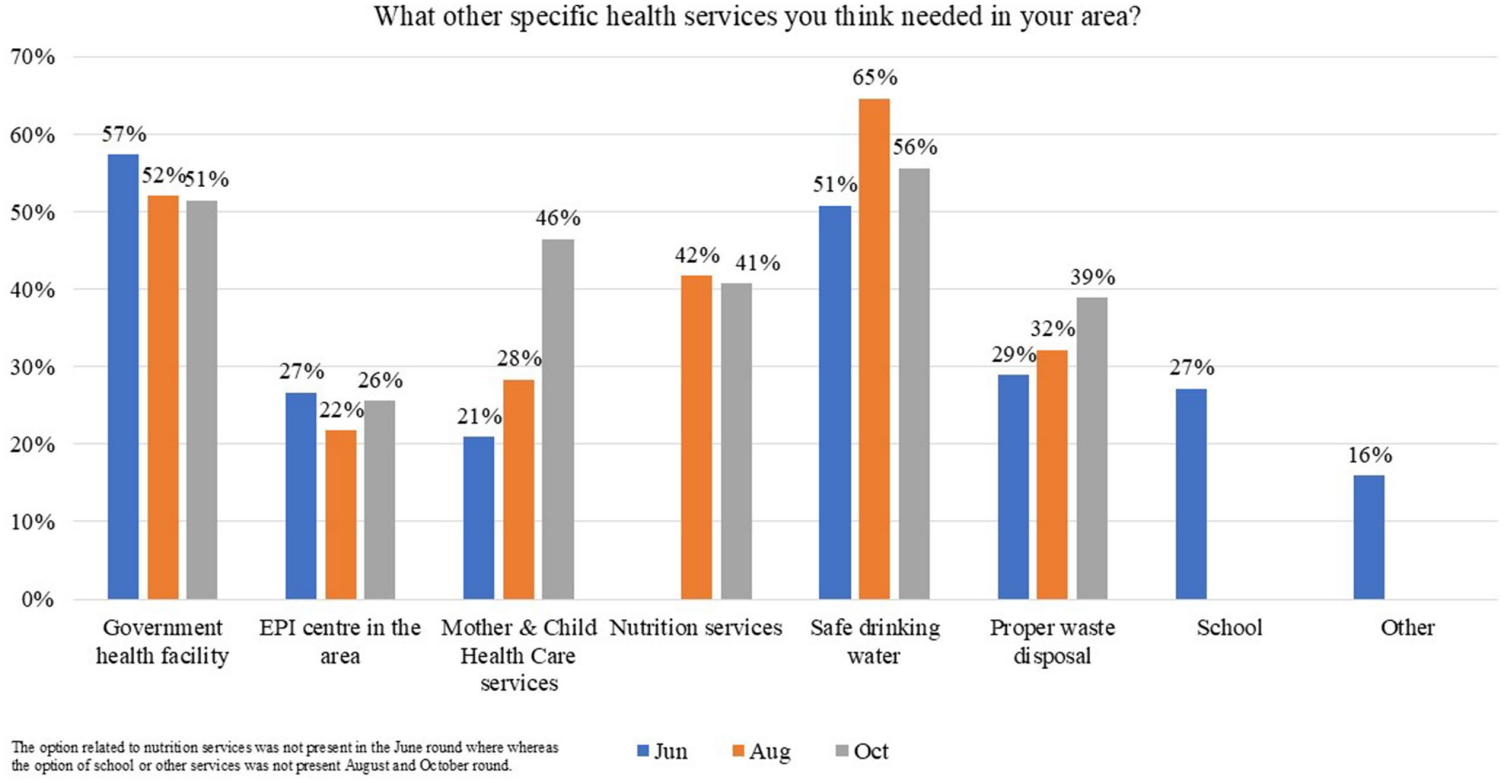
Participants’ responses on what other specific services are needed in the area in Karachi, Sindh, Pakistan.

Overall, 772 (88.1%) of participants were satisfied with staff behavior at health camps in June and 324 (88.3%) in August 2021, whereas 353 (92.2%) satisfaction was reported in October 2021. Almost all participants reported that health camps were useful and should be organized again. Participants’ main requests were to provide consultant medical doctors/specialists, i.e., pediatricians [380 (43.3%) in June, 173 (47.1%) in August, and 272 (71.0%) in October], Ear, Nose, and Throat (ENT) specialists [271 (30.9%) in June, 181 (49.3%) in August, and 113 (29.5%) in October], and dermatologists [307 (35.0%) in June, 203 (55.3%) in August, and 187 (48.8%) in October] in future health camps (Figure 2). The other services requested in the survey include a government health facility [503 (57.4%) in June, 191 (52.0%) in August, and 197 (51.4%) in October], provision of safe drinking water [445 (50.8%) in June, 237 (64.6%) in August, and 213 (55.6%) in October], proper waste disposal [253 (28.9%) in June, 118 (32.2%) in August, and 149 (38.9%) in October], as specified by the participants (Figure 3).

In all three rounds, 82–95% of participants who reported not taking their child to the nearest health facility for routine immunization also received vaccination services at the health camps. At health camps, 69 (82.1%) parents who did not take their children to the nearest health facility for vaccination agreed to vaccinate their children in June (*p*-values = 0.673), 26 (89.7%) in August (*p*-values = 0.523), and 18 (94.7%) in October (*p*-values = 0.782). Among those parents who do not allow their children to receive OPV from polio teams at their doorstep, 48 (81.4%) vaccinated children at the health camps in June (*p*-values = 0.822), eight (80.0%) vaccinated their children in August (*p*-values = 0.599), and 13 (86.7%) did so in October (*p*-values = 0.062). However, this result did not show any evidence of statistically significant association based on Pearson chi-square *p*-values as shown in Table 3.

**Table 3:** Status of vaccination of children at health camps who did not receive polio vaccine from routine immunization program and through polio teams at households.

Variables	The child received polio vaccine at a health camp					
	June 2021		August 2021		October 2021	
	Yes	No	Yes	No	Yes	No
Parents take the child to the nearest health facility for routine EPI vaccination						
Yes	370 (79.9%)	90 (20.1%)	226 (85.3%)	39 (14.7%)	290 (96.0%)	12 (4.0%)
No	69 (82.1%)	15 (17.9%)	26 (89.7%)	3 (10.3%)	18 (94.7%)	1 (5.3%)
*p*-value	0.673		0.523		0.782	
Parent allows their child to get oral polio vaccine from polio teams at their doorstep						
Yes	391 (80.1%)	97 (19.9%)	224 (85.9%)	40 (14.1%)	295 (96.4%)	11 (3.6%)
No	48 (81.4%)	11 (18.6%)	8 (80.0%)	2 (20.0%)	13 (86.7%)	2 (13.3%)
*p*-value	0.822		0.599		0.062	

UC level analysis showed a cumulative 14.5% reduction in persistently missed children (PMCs) in June 2021 SNIDs as compared to the March 2021 NIDs in all SHRUCs. Similarly, zero-dose children also decreased by 4.6% during June 2021 SNIDs as compared to March 2021 NIDs in SHRUCs.

Results showed an overall 10.3% reduction in PMCs in June SNIDs as compared to March NIDs in areas with health camps. Overall, from a total of 55 areas where health camps were organized during June SNIDs, still missed children who were not available at the time of the campaign reduced in 8 (14.5%) areas, still refusal reduced in 25 (45.5%) areas, and total still missed children reduced in 8 (14.5%) areas. Persistently missed children were reduced in 38 (69.1%) areas and zero-doze children were reduced in 8 (14.5%) areas in June SNIDs as compared to March NIDs in 2021. Reductions in clusters of still not available children, still refusals, persistently missed children, and zero dose children were observed in areas where health camps were conducted in all three rounds of health camps (Supplementary Table 1 and Tables 2, 3).

## Discussion

Health camps are organized to provide basic health services in underprivileged communities as evidenced by previous literature (12). They provided integrated services through outreach camps placed in marginalized communities to improve polio vaccine acceptability and reach among children who were missed during polio SIAs in high-risk areas of Pakistan (9). We explored the effectiveness of organizing health camps in high-risk areas of Karachi, Sindh, Pakistan. Despite polio vaccine hesitancy in high-risk communities of Karachi, the positive collective impact of community mobilization and the delivery of maternal and child health services and immunizations through health camps during and after supplementary immunization activities was evident. Additionally, health camps effectively increased polio vaccine coverage and were deemed feasible and acceptable by the community.

We identified that, during three rounds of health camps, more than 80% of participants who did not take their children to the nearest health facility for routine immunization agreed to have their children vaccinated at health camps. Health camps were effective in improving routine immunization and OPV vaccination coverage as also observed in a previous study conducted in the high-risk areas of Karachi, Sindh province, Balochistan province, and Khyber Pakhtunkhwa province in Pakistan (13). Previous studies conducted in urban slums indicate that Pashtun ethnicity, distance to vaccination centers, lack of mother’s education, and low household income were factors associated with low vaccination coverage (14, 15). Our study observed that health camps appeal more to parents and caregivers who did not visit the nearest government health facility to vaccinate their children but attended health camps for vaccination and other health services. One of the most likely explanations to this finding could be the environment of the health camps and social mobilization in the camps, as our study showed that the majority of participants were afraid of side effects, not aware of the nearby health facility, and visiting the private health facilities for routine immunization as well as polio vaccination.

Similarly, more than 80% of participants who did not accept OPV from polio teams at their doorstep also allowed their children to receive polio vaccines at the health camps. Although not statistically significant, these results show that these parents may not accept polio drops in their homes due to mistrust of the quality of vaccines, fear of side effects, and negative social media videos about polio vaccines (16) were willing to vaccinate their children during health camps. So it means they do not necessarily have an issue with vaccines or vaccination but with repeated visits to their homes by polio teams.

Further, most parents who were not in favor of OPV being administered by polio teams at their doorsteps preferred to get their children vaccinated at health camps.

In areas where health camps were conducted, there was a notable increase in the coverage of children previously missed in polio campaigns. Additionally, a marked decrease was observed in the number of non-attendees, refusals, persistently missed children, and zero-dose children. So, it is pertinent that health camps can play a contributing role in addressing the community’s acceptance of vaccination. This indicates an extended impact of the health camp intervention in addition to serving the purpose of improving the reputation of the polio program and building trust between the polio program and underserved communities of high-risk areas in Karachi, as observed in previous studies (9, 12, 13, 17). The health camps initiative played a crucial role in improving the reputation of the polio program and establishing trust among underserved communities in Karachi’s high-risk areas. They provided a unique opportunity for direct engagement with the community, particularly with female household members (18). This interaction allowed for a deeper understanding of the challenges faced in seeking healthcare for mothers and children. Additionally, it was an avenue to identify core social needs and required services in these areas (18, 19).

We have found that community perception for polio, as a harmful disease, is very low in the areas where a high number of children were found missed in the polio campaigns. Therefore, the Participants who were attending the health camps had low awareness of how poliovirus is transmitted or appropriate prevention measures are available. This lack of knowledge or low-risk perception toward polio could be a factor in refusing polio vaccination in these areas. These findings support other studies conducted on the same subject (20, 21).

There are a few limitations of this study. The study was biased purposively to those areas which have a high number of still missed children. Therefore there are chances that health camps can misjudge the reasons of the children who are refusing the polio vaccination. More studies are needed to find reasons for the missed children with the random sampling technique. Health camp locations changed as per request of the district EOCs, as well as security challenges and other issues. Also, health camps organized at the provincial level, in coordination with district-level management teams, focused on areas with a high incidence of children that were still missed during polio campaigns, which could potentially bias the results of the study. Despite these limitations, the ability to reach and vaccinate significant numbers of persistently missed children in high-risk areas through health camps is a major strength of this study.

The findings of this study underscore the importance of continuing to organize health camps in underserved, high-risk areas of Karachi to further enhance the reputation of the polio program. Additionally, there is a need for responsiveness to community demands for the inclusion of specialist healthcare services, such as pediatricians, otolaryngologist, and dermatologists, in future health camps. The study also highlights requests by communities that participated in the survey for broader public services, including government health facilities, access to safe drinking water, efficient waste disposal systems, comprehensive maternal and child healthcare services, and immunization centers in their areas. These interventions will not only improve the overall health and wellbeing of underserved populations in these areas, they will also help to build trust and improve the standing of the polio program amongst these communities.

## Data Availability

The original contributions presented in the study are included in the article/Supplementary material, further inquiries can be directed to the corresponding author.

## References

[1] World Health Organization. A crippling and life-threatening disease. (2022). Available at: https://www.who.int/news-room/spotlight/history-of-vaccination/history-of-polio-vaccination?topicsurvey=ht7j2q)andgad_source=1andgclid=CjwKCAjw1emzBhB8EiwAHwZZxWlSk2RCIAyeQhMnFpCzoxG44bo2wi3t0U3doNQ5fhTPL4aYcVqaWxoC7o0QAvD_BwE (Accessed December 16, 2024).

[2] World Health Organization. Poliomyelitis. (2023). Available at: https://www.who.int/news-room/fact-sheets/detail/poliomyelitis (Accessed December 16, 2024).

[3] Mehndiratta MM Mehndiratta P Pande R. Poliomyelitis: historical facts, epidemiology, and current challenges in eradication. Neurohospitalist. (2014) 4:223–9. doi: 10.1177/1941874414533352, PMID: 25360208 PMC4212416

[4] World Health Organization. Two out of three wild poliovirus strains eradicated. (2019). Available at: https://www.who.int/news-room/feature-stories/detail/two-out-of-three-wild-poliovirus-strains-eradicated (Accessed December 16, 2024).

[5] Global Polio Eradication Initiative. Polio this week. (2024). Available at: https://polioeradication.org/about-polio/polio-this-week/ (Accessed December 16, 2024).

[6] Mbaeyi C Haq A Safdar RM Khan Z Corkum M Henderson E. Progress toward poliomyelitis eradication — Pakistan, January 2023–June 2024. Morb Mortal Wkly Rep. (2024) 73:788–92. 10.15585/mmwr.mm7336a2 PMC11392225 39264848

[7] Global Polio Eradication Initiative. Meeting of the technical advisory group (TAG) on polio eradication in Pakistan. February 2021. (2021). Available at: https://polioeradication.org/wp-content/uploads/2021/04/Pakistan-TAG-Report-20210209-11.pdf (Accessed September 18, 2024).

[8] Trust for Vaccines and Immunization. Project Health camps to increase participation in SIAs in Pakistan. August to December 2021. (2021). Available at: https://tvi.org.pk/health-camps-in-super-high-risk-and-high-risk-ucs-of-pakistan/ (Accessed September 18, 2024).

[9] National Emergency Operation Centre, Islamabad. Pakistan polio eradication initiative: National Emergency Action Plan 2021–2023. (2022). Available at: https://polioeradication.org/wp-content/uploads/2022/02/NEAP-2021-2023.pdf (Accessed September 18, 2024).

[10] Global Polio Eradication Initiative. Integration is one of five key strategic objectives in the 2022–2026 polio eradication strategy. (2029). Available at: https://polioeradication.org/who-we-are/polio-endgame-strategy-2019-2023/ (Accessed September 18, 2024).

[11] Abbasi FH Shaikh AA Mehraj J Raza SM Rasool S Bullo UF. Vaccine hesitancy and perceptions of the community about polio in high-risk areas of Karachi, Sindh, Pakistan. Vaccine. (2022) 11:70. doi: 10.3390/vaccines11010070, PMID: 36679915 PMC9866813

[12] Habib MA Soofi S Cousens S Anwar S ul Haque N Ahmed I. Community engagement and integrated health and polio immunisation campaigns in conflict-affected areas of Pakistan: a cluster randomised controlled trial. Lancet Glob Health. (2017) 5:e593–603. doi: 10.1016/S2214-109X(17)30184-5, PMID: 28495264 PMC5439031

[13] Habib MA Soofi SB Hussain Z Ahmed I Tahir R Anwar S. A holistic strategy of mother and child health care to improve the coverage of routine and polio immunization in Pakistan: results from a demonstration project. Vaccine. (2024) 12:89. doi: 10.3390/vaccines12010089, PMID: 38250902 PMC10819799

[14] Habib SS Zaidi S Riaz A Tahir HN Mazhar LA Memon Z. Social determinants of low uptake of childhood vaccination in high-risk squatter settlements in Karachi, Pakistan–a step towards addressing vaccine inequity in urban slums. Vaccine. (2024) 17:100427. doi: 10.1016/j.jvacx.2023.100427, PMID: 38299204 PMC10827488

[15] Soofi SB Vadsaria K Mannan S Habib MA Tabassum F Hussain I. Factors associated with vaccine refusal (polio and routine immunization) in high-risk areas of Pakistan: a matched case-control study. Vaccine. (2023) 11:947. doi: 10.3390/vaccines11050947, PMID: 37243051 PMC10222589

[16] Bullo UF Mehraj J Raza SM Rasool S Ansari NN Shaikh AA. An experience of mass administration of fractional dose inactivated polio vaccine through intradermal needle-free injectors in Karachi, Sindh, Pakistan. BMC Public Health. (2021) 21:1–7. doi: 10.1186/s12889-020-10041-8 33407294 PMC7789602

[17] Garon JR Orenstein WA. Overcoming barriers to polio eradication in conflict areas. Lancet Infect Dis. (2015) 15:1122–4. doi: 10.1016/S1473-3099(15)00008-0, PMID: 26179315

[18] Das JK Khan A Tabassum F Padhani ZA Habib A Mirani M. The last mile—community engagement and conditional incentives to accelerate polio eradication in Pakistan: study protocol for a quasi-experimental trial. Methods Protoc. (2023) 6:83. doi: 10.3390/mps6050083, PMID: 37736966 PMC10514870

[19] Sodhar IA Hussaini AS Brown MJ. Eradicating polio: a perspective from Pakistan. Trop Med Int Health. (2023) 28:839–43. doi: 10.1111/tmi.13935, PMID: 37775966

[20] Habib MA Tabassum F Hussain I Khan TJ Syed N Shaheen F. Exploring knowledge and perceptions of polio disease and its immunization in polio high-risk areas of Pakistan. Vaccine. (2023) 11:1206. doi: 10.3390/vaccines11071206, PMID: 37515022 PMC10386680

[21] Khan MU Ahmad A Aqeel T Salman S Ibrahim Q Idrees J. Knowledge, attitudes and perceptions towards polio immunization among residents of two highly affected regions of Pakistan. BMC Public Health. (2015) 15:1100. doi: 10.1186/s12889-015-2471-1, PMID: 26541976 PMC4635542

