# Supplementary material for: Community acceptance of services and effectiveness of health camps in high-risk areas of Karachi, Sindh, Pakistan, 2021
Source: Front Public Health. 2025 Jan 8;12:1498016. doi: 10.3389/fpubh.2024.1498016 (PMC11751023; doi:10.3389/fpubh.2024.1498016)
Supplement: Supplementary file 1 [file Data_Sheet_1.docx]

**Health camps effectiveness assessment**

Health camps location: **_____________________________________** District: ________________________________

UC: ____________________________ Area/ Mohalla/Locality within UC: __________________________________

Date of Survey: ______________ Name of Surveyor: __________________ Designation: _______________________

****Important: Please take interview after respondent availed the services and about to leave the camp site.***

| **S. No.** | **Questions** | **Possible Answers** |
| --- | --- | --- |
| 1 | Gender of the Respondent | 1. Male 2. Female |
| 2 | Age of the Respondent | ------------------------- in years |
| 3 | How did you hear about the health camp? | 1. Announcements (Mosque, mobile miking)  2. LHW  3. Polio Team  4. Neighbour  5. Other (specify) ---------------------------------------- |
| 4 | Distance of Health camp from your Home? | Km: _______________________  Meters: ____________________ |
| 5 | How much time is spent to arrive at this campsite from your home? (by walk) | 1. 5 min 2. 10-15 min 3. 20 -25 min 4. >30 min |
| 6 a | Do you have children?  (if **no** then skip Questions **6 b and 10 a – 11 c**) | 1. Yes 2. No |
| 6 b | If yes,  Please specify number of Children in each age category | 1. _______________< 1 year 2. _______________ 1- < 5 years 3. _______________ 6 – 14 years 4. _______________ Above 14 years |
| 7 | How many family members come along with you to avail of the services here? | ----------------------- number of family members |
| 8 | Which services did you avail of at this health camp? | 1. Childhood vaccination ________ 2. Pregnant women vaccination ________ 3. Nutrition support ________ 4. Consultation with doctor ________ 5. Other (specify)_________________________ |
| 9 | How important are vaccinations for you or your child? | 1. Very important 2. Important 3. Not important |
| 10 a | Did your child receive any vaccination here at health camps? | 1. Yes 2. No 3. Not applicable |
| 10 b | If yes,  check card and mark in the provided space for vaccine type | **At birth =** BCG ___, OPV 0___,  **6 weeks =** Pentavalent 1___, PCV 1 ___, OPV1 ___, Rota 1 ___,  **10 weeks =** Pentavalent 2___, PCV 2 ___, OPV2 ___, Rota 2 ___,  **14 weeks** = Pentavalent 3___, PCV 3___, OPV3 ___,  IPV 1 ___,  **9 months =** Measles 1 ___, TCV ___, IPV 2 ___,  **15 months =** Measles 2 ___, |
| 10 c | Do you take your child to the nearest health facility for routine EPI vaccination? | 1. Yes 2. No |
| 10 d | If No,  Why do you not prefer routine vaccination of your children? | 1. Don’t know about routine vaccination. 2. Don’t know about nearest EPI facility. 3. Prefer private vaccination. 4. Not sure about vaccine quality 5. Afraid of side effects 6. Misconception/ community rumour 7. Other (specify) ________________________ |
| 11 a | Do you allow your child to get oral polio vaccine from polio teams at your doorstep? | 1. Yes 2. No |
| 11 b | If No,  Where do you prefer to get OPV vaccination for your child? | 1. Do not prefer the polio vaccine. 2. From government health facility 3. From private health facility 4. From local general practitioner 5. Other (specify)_________________________ |
| 11 c | If No,  Why do you not prefer the polio vaccination of your child? | 1. Religious 2. Not sure about content of vaccine 3. Polio team’s behaviour 4. Repeated visits 5. Private Doctor’s advice 6. Other (specify)_______________________ |
| 12 | Do you know, what happens if a child gets Polio? | 1. Paralysis 2. Death 3. Don't know. 4. Other (specify)_______________________ |
| 13 | What do you think about how polio is transmitted in children? | 1. Contaminated water 2. Contaminated food 3. Poor hygiene 4. not vaccinated 5. Don't know. 6. Other (specify)_______________________ |
| 14 | How polio can be prevented? | 1. Oral Polio vaccine (OPV) - drops 2. Inactivated Polio vaccine (IPV) - injection 3. Don't know. 4. Other (specify)_______________________ |
| 15 | Are you satisfied with the services that you have received at this health camp? | 1. Satisfied 2. Partially satisfied. 3. Not satisfied |
| 16 | Are you satisfied with the medicines that were provided to you at the health camp? | 1. Satisfied 2. Partially satisfied. 3. Not satisfied |
| 17 | Do you think these camps are useful and should be happened again? | 1. Yes 2. No |
| 18 | Are you satisfied with the service providers' (staff) behavior at the Health Camp? | 1. Satisfied 2. Partially satisfied. 3. Not satisfied |
| 19 | What other health services should be provided at the health camps (in your opinion)? | 1. Paediatrician (Consultant) 2. ENT specialist (eye, nose throat) 3. Skin specialist 4. Infectious Disease specialist 5. Laboratory test 6. Other (specify)__________________________ |
| 20 | What other specific health services you think needed in your area? | 1. Government health facility 2. EPI centre in the area 3. MNCH centre 4. Safe drinking water 5. Proper waste disposal 6. School 7. Other (specify)__________________________ |
| 21 | What services do you prefer to avail here as compared to the nearest Government Health facility? | 1. Vaccines 2. Consultation with Medical Doctor 3. Availability of free medicines 4. Other (specify)__________________________ |
| 22 | Any other comments:  ----------------------------------------------------------------------------------------------------------------------  ---------------------------------------------------------------------------------------------------------------------- | |

*****Important: Please pay gratitude to the participant for his/ her time and valuable feedback.***

***Thank You!!***
