# Supplementary material for: Community acceptance of services and effectiveness of health camps in high-risk areas of Karachi, Sindh, Pakistan, 2021
Source: Front Public Health. 2025 Jan 8;12:1498016. doi: 10.3389/fpubh.2024.1498016 (PMC11751023; doi:10.3389/fpubh.2024.1498016)

**Supplementary tables and figures**

Supplementary table 1a. Union councils level Impact of health camps in super high-risk union councils of Karachi in June 2021.

| **SHRUCs** | **Mar 21 Still NA** | **Jun 21 Still NA** | **% of change** | **Mar 21 Still Refusal** | **Jun 21 Still Refusal** | **% of change** | **Mar 21 Total Still Missed** | **Jun 21 Total Still Missed** | **% of change** | **Mar 21 PMC 3** | **Jun 21 PMC 3** | **% of change** | **Mar 21 Still Zero Dose** | **Jun 21 Still Zero Dose** | **% of change** |
| --- | --- | --- | --- | --- | --- | --- | --- | --- | --- | --- | --- | --- | --- | --- | --- |
| **CHISTI NAGAR-7** | 271 | 476 | 75.6% | 398 | 548 | 37.7% | 669 | 1024 | 53.1% | 185 | 162 | -12.4% | 31 | 60 | 93.5% |
| **GUJRO A** | 298 | 483 | 62.1% | 274 | 326 | 19.0% | 572 | 809 | 41.4% | 129 | 114 | -11.6% | 267 | 90 | -66.3% |
| **GUJRO B** | 336 | 618 | 83.9% | 493 | 551 | 11.8% | 829 | 1169 | 41.0% | 184 | 173 | -6.0% | 419 | 134 | -68.0% |
| **GUJRO C** | 1043 | 1629 | 56.2% | 364 | 376 | 3.3% | 1407 | 2005 | 42.5% | 169 | 139 | -17.8% | 422 | 287 | -32.0% |
| **GUJRO D** | 1002 | 1186 | 18.4% | 367 | 354 | -3.5% | 1369 | 1540 | 12.5% | 158 | 116 | -26.6% | 335 | 244 | -27.2% |
| **GUJRO E** | 671 | 1193 | 77.8% | 370 | 418 | 13.0% | 1041 | 1611 | 54.8% | 133 | 122 | -8.3% | 491 | 294 | -40.1% |
| **ISLIMIA COLONY-9** | 600 | 948 | 58.0% | 625 | 582 | -6.9% | 1225 | 1530 | 24.9% | 287 | 249 | -13.2% | 96 | 184 | 91.7% |
| **ITTEHAD TOWN-2** | 840 | 1230 | 46.4% | 958 | 994 | 3.8% | 1798 | 2224 | 23.7% | 449 | 360 | -19.8% | 215 | 276 | 28.4% |
| **MANGOPIR-8** | 766 | 1468 | 91.6% | 995 | 1265 | 27.1% | 1761 | 2733 | 55.2% | 321 | 301 | -6.2% | 161 | 197 | 22.4% |
| **MUSLIMABAD-2** | 435 | 680 | 56.3% | 636 | 572 | -10.1% | 1071 | 1252 | 16.9% | 296 | 281 | -5.1% | 510 | 635 | 24.5% |
| **MUZAFARABAD-1** | 718 | 1029 | 43.3% | 902 | 806 | -10.6% | 1620 | 1835 | 13.3% | 454 | 397 | -12.6% | 567 | 859 | 51.5% |
| **SONGAL-5** | 1793 | 2721 | 51.8% | 1181 | 1289 | 9.1% | 2974 | 4010 | 34.8% | 550 | 421 | -23.5% | 348 | 425 | 22.1% |
| **Grand Total** | **8773** | **13661** | **55.7%** | **7563** | **8081** | **6.8%** | **16336** | **21742** | **33.1%** | **3315** | **2835** | **-14.5%** | **3862** | **3685** | **-4.6%** |

Reduction in still missed children, not available children, refusal, persistently missed children, and zero dose children in the current campaign round as compared to previous round

Increase in still missed children, not available children, refusal, persistently missed children, and zero dose children in the current campaign round as compared to previous round

super high-risk

and high-risk union councils

Supplementary table 1b. Area level impact of health camps in super high risk union councils of Karachi in June 2021.

| **District** | **Town** | **UC** | **AS Name** | NID MARCH 2021 | | | | | SNID JUNE 2021 | | | | | % of Change | | | | |
| --- | --- | --- | --- | --- | --- | --- | --- | --- | --- | --- | --- | --- | --- | --- | --- | --- | --- | --- |
|  |  |  |  | **Still NA** | **Still Refusal** | **Total Still Missed** | **PMC 3** | **Still Zero Dose** | **Still NA** | **Still Refusal** | **Total Still Missed** | **PMC 3** | **Still Zero Dose** | **Still NA** | **Still Refusal** | **Total Still Missed** | **PMC 3** | **Still Zero Dose** |
| EAST | GADAP | GUJRO A | AIMAN | 74 | 39 | 113 | 21 | 93 | 114 | 47 | 161 | 24 | 32 | 54% | 21% | 42% | 14% | -66% |
| EAST | GADAP | GUJRO A | ROSHILA | 30 | 27 | 57 | 12 | 28 | 52 | 50 | 102 | 11 | 4 | 73% | 85% | 79% | -8% | -86% |
| EAST | GADAP | GUJRO B | AZAM BIBI | 39 | 74 | 113 | 28 | 93 | 83 | 82 | 165 | 27 | 21 | 113% | 11% | 46% | -4% | -77% |
| EAST | GADAP | GUJRO C | ANUM | 33 | 14 | 47 | 8 | 4 | 119 | 12 | 131 | 7 | 12 | 261% | -14% | 179% | -13% | 200% |
| EAST | GADAP | GUJRO C | AYESHA BANO | 16 | 9 | 25 | 1 | 29 | 108 | 16 | 124 | 2 | 22 | 575% | 78% | 396% | 100% | -24% |
| EAST | GADAP | GUJRO D | TAZEEN | 75 | 35 | 110 | 16 | 14 | 108 | 24 | 132 | 10 | 16 | 44% | -31% | 20% | -38% | 14% |
| EAST | GADAP | GUJRO E | ERUM SHAHEEN | 66 | 28 | 94 | 9 | 29 | 144 | 22 | 166 | 11 | 9 | 118% | -21% | 77% | 22% | -69% |
| KAMARI | BALDIA | ITTEHAD TOWN-2 | AZRA PARVEEN | 49 | 31 | 80 | 17 | 8 | 62 | 28 | 90 | 14 | 16 | 27% | -10% | 13% | -18% | 100% |
| KAMARI | BALDIA | ITTEHAD TOWN-2 | MAREENA | 68 | 90 | 158 | 51 | 5 | 80 | 89 | 169 | 44 | 6 | 18% | -1% | 7% | -14% | 20% |
| KAMARI | BALDIA | ITTEHAD TOWN-2 | PALWASHA | 37 | 59 | 96 | 17 | 7 | 104 | 69 | 173 | 18 | 12 | 181% | 17% | 80% | 6% | 71% |
| KAMARI | BALDIA | ITTEHAD TOWN-2 | SAWERA | 43 | 58 | 101 | 27 | 11 | 32 | 76 | 108 | 25 | 11 | -26% | 31% | 7% | -7% | 0% |
| KAMARI | BALDIA | ITTEHAD TOWN-2 | SHAMIM NIZAM | 63 | 50 | 113 | 14 | 7 | 61 | 45 | 106 | 17 | 13 | -3% | -10% | -6% | 21% | 86% |
| KAMARI | BALDIA | ITTEHAD TOWN-2 | SIDRA | 13 | 29 | 42 | 14 | 9 | 31 | 30 | 61 | 12 | 20 | 138% | 3% | 45% | -14% | 122% |
| KAMARI | BALDIA | ITTEHAD TOWN-2 | UROOJ | 46 | 29 | 75 | 12 | 10 | 68 | 25 | 93 | 8 | 13 | 48% | -14% | 24% | -33% | 30% |
| MALIR | LANDHI | MUSLIMABAD-2 | AMNA SALEEM | 32 | 62 | 94 | 25 | 30 | 53 | 39 | 92 | 27 | 37 | 66% | -37% | -2% | 8% | 23% |
| MALIR | LANDHI | MUSLIMABAD-2 | HINA MAQSOOD | 51 | 17 | 68 | 14 | 53 | 65 | 16 | 81 | 9 | 64 | 27% | -6% | 19% | -36% | 21% |
| MALIR | LANDHI | MUSLIMABAD-2 | KOUSAR | 23 | 50 | 73 | 23 | 42 | 28 | 50 | 78 | 25 | 49 | 22% | 0% | 7% | 9% | 17% |
| MALIR | LANDHI | MUSLIMABAD-2 | REEMA KHAN | 31 | 55 | 86 | 34 | 45 | 63 | 77 | 140 | 28 | 70 | 103% | 40% | 63% | -18% | 56% |
| MALIR | LANDHI | MUSLIMABAD-2 | RUKHSANA ANWER | 36 | 65 | 101 | 29 | 18 | 31 | 59 | 90 | 31 | 25 | -14% | -9% | -11% | 7% | 39% |
| MALIR | LANDHI | MUSLIMABAD-2 | SAMRA KAREEM | 12 | 52 | 64 | 24 | 57 | 69 | 42 | 111 | 18 | 62 | 475% | -19% | 73% | -25% | 9% |
| MALIR | LANDHI | MUSLIMABAD-2 | ZAHIDA | 16 | 53 | 69 | 31 | 9 | 35 | 40 | 75 | 24 | 11 | 119% | -25% | 9% | -23% | 22% |
| MALIR | LANDHI | MUZAFARABAD-1 | NEELAM | 55 | 50 | 105 | 27 | 41 | 51 | 46 | 97 | 25 | 54 | -7% | -8% | -8% | -7% | 32% |
| MALIR | LANDHI | MUZAFARABAD-1 | NUSRAT | 39 | 96 | 135 | 55 | 50 | 61 | 104 | 165 | 52 | 65 | 56% | 8% | 22% | -5% | 30% |
| MALIR | LANDHI | MUZAFARABAD-1 | SAIMA BIBI | 40 | 51 | 91 | 27 | 48 | 61 | 54 | 115 | 31 | 51 | 53% | 6% | 26% | 15% | 6% |
| MALIR | LANDHI | MUZAFARABAD-1 | SAIMA HAMEED | 41 | 80 | 121 | 31 | 14 | 65 | 60 | 125 | 23 | 44 | 59% | -25% | 3% | -26% | 214% |
| MALIR | LANDHI | MUZAFARABAD-1 | SHUMAILA ANJUM | 56 | 63 | 119 | 26 | 59 | 81 | 82 | 163 | 24 | 79 | 45% | 30% | 37% | -8% | 34% |
| MALIR | LANDHI | MUZAFARABAD-1 | TANZILA | 48 | 62 | 110 | 28 | 55 | 77 | 60 | 137 | 29 | 66 | 60% | -3% | 25% | 4% | 20% |
| MALIR | LANDHI | MUZAFARABAD-1 | ZUBARIYA | 23 | 69 | 92 | 35 | 43 | 48 | 52 | 100 | 30 | 74 | 109% | -25% | 9% | -14% | 72% |
| WEST | GADAP | MANGOPIR-8 | ALISHBA | 28 | 37 | 65 | 16 | 6 | 71 | 66 | 137 | 22 | 9 | 154% | 78% | 111% | 38% | 50% |
| WEST | GADAP | MANGOPIR-8 | ALIYA MAZHAR | 39 | 54 | 93 | 22 | 3 | 52 | 62 | 114 | 20 | 8 | 33% | 15% | 23% | -9% | 167% |
| WEST | GADAP | MANGOPIR-8 | AZRA SANA | 36 | 4 | 40 |  | 10 | 60 | 7 | 67 |  | 18 | 67% | 75% | 68% | #DIV/0! | 80% |
| WEST | GADAP | MANGOPIR-8 | QAIM ALI | 18 | 26 | 44 | 9 | 4 | 53 | 22 | 75 | 6 | 8 | 194% | -15% | 70% | -33% | 100% |
| WEST | GADAP | MANGOPIR-8 | RABIA | 35 | 9 | 44 | 3 | 7 | 59 | 17 | 76 | 2 | 11 | 69% | 89% | 73% | -33% | 57% |
| WEST | GADAP | MANGOPIR-8 | ZARQA FAIZ | 16 | 47 | 63 | 16 | 1 | 25 | 58 | 83 | 19 | 2 | 56% | 23% | 32% | 19% | 100% |
| WEST | GADAP | SONGAL-5 | FATIMA AMEER | 43 | 35 | 78 | 16 | 1 | 58 | 46 | 104 | 15 | 10 | 35% | 31% | 33% | -6% | 900% |
| WEST | GADAP | SONGAL-5 | NAZIA HUSSAIN | 41 | 1 | 42 | 4 | 19 | 33 | 1 | 34 | 2 | 10 | -20% | 0% | -19% | -50% | -47% |
| WEST | GADAP | SONGAL-5 | SHAZIA | 24 | 15 | 39 | 9 | 4 | 44 | 9 | 53 | 2 | 18 | 83% | -40% | 36% | -78% | 350% |
| WEST | GADAP | SONGAL-5 | SIDRA | 38 | 23 | 61 | 6 | 4 | 77 | 33 | 110 | 8 | 14 | 103% | 43% | 80% | 33% | 250% |
| WEST | GADAP | SONGAL-5 | SULTANA | 88 | 83 | 171 | 27 | 29 | 152 | 71 | 223 | 21 | 26 | 73% | -14% | 30% | -22% | -10% |
| WEST | GADAP | SONGAL-5 | TEHMINA | 33 | 4 | 37 | 1 | 37 | 72 | 5 | 77 |  | 39 | 118% | 25% | 108% | -100% | 5% |
| WEST | GADAP | SONGAL-5 | ZUBAIDA | 53 | 5 | 58 | 7 | 12 | 28 | 2 | 30 | 1 | 5 | -47% | -60% | -48% | -86% | -58% |
| WEST | ORANGI | CHISTI NAGAR-7 | ALMAS | 29 | 22 | 51 | 9 | 1 | 44 | 28 | 72 | 11 | 1 | 52% | 27% | 41% | 22% | 0% |
| WEST | ORANGI | CHISTI NAGAR-7 | DILNAZ | 11 | 8 | 19 | 7 | 3 | 20 | 12 | 32 | 3 | 8 | 82% | 50% | 68% | -57% | 167% |
| WEST | ORANGI | CHISTI NAGAR-7 | MARIA SIDDIQUI | 17 | 39 | 56 | 14 | 2 | 34 | 44 | 78 | 12 | 1 | 100% | 13% | 39% | -14% | -50% |
| WEST | ORANGI | CHISTI NAGAR-7 | MARIA YAMEEN | 15 | 17 | 32 | 9 | 2 | 36 | 41 | 77 | 11 | 3 | 140% | 141% | 141% | 22% | 50% |
| WEST | ORANGI | CHISTI NAGAR-7 | NOSHABA | 18 | 50 | 68 | 20 |  | 25 | 69 | 94 | 13 | 1 | 39% | 38% | 38% | -35% | #DIV/0! |
| WEST | ORANGI | CHISTI NAGAR-7 | SHAHNAZ FATIMA | 48 | 47 | 95 | 33 | 3 | 42 | 59 | 101 | 31 | 16 | -13% | 26% | 6% | -6% | 433% |
| WEST | ORANGI | CHISTI NAGAR-7 | SHANZA RANI | 22 | 25 | 47 | 7 | 5 | 71 | 30 | 101 | 8 | 5 | 223% | 20% | 115% | 14% | 0% |
| WEST | SITE | ISLIMIA COLONY-9 | AMBREEN | 50 | 27 | 77 | 12 | 4 | 76 | 25 | 101 | 8 | 9 | 52% | -7% | 31% | -33% | 125% |
| WEST | SITE | ISLIMIA COLONY-9 | AYESHA | 23 | 29 | 52 | 14 | 9 | 46 | 18 | 64 | 12 | 9 | 100% | -38% | 23% | -14% | 0% |
| WEST | SITE | ISLIMIA COLONY-9 | BAKHT BIBI | 32 | 61 | 93 | 33 | 12 | 42 | 62 | 104 | 31 | 21 | 31% | 2% | 12% | -6% | 75% |
| WEST | SITE | ISLIMIA COLONY-9 | FATIMA ANJUM | 58 | 40 | 98 | 28 | 2 | 54 | 30 | 84 | 17 | 2 | -7% | -25% | -14% | -39% | 0% |
| WEST | SITE | ISLIMIA COLONY-9 | JAMILA | 32 | 73 | 105 | 29 | 13 | 34 | 59 | 93 | 28 | 26 | 6% | -19% | -11% | -3% | 100% |
| WEST | SITE | ISLIMIA COLONY-9 | NAZNEEN | 48 | 63 | 111 | 21 | 6 | 72 | 68 | 140 | 18 | 10 | 50% | 8% | 26% | -14% | 67% |
| WEST | SITE | ISLIMIA COLONY-9 | UROOJ NAZ | 53 | 69 | 122 | 37 | 2 | 79 | 47 | 126 | 28 | 12 | 49% | -32% | 3% | -24% | 500% |
|  |  |  |  |  |  |  |  |  |  |  |  |  |  |  |  |  |  |  |
| Grand total | | | | 2103 | 2310 | 4413 | 1065 | 1112 | 3413 | 2387 | 5800 | 955 | 1260 | 62% | 3% | 31% | -10% | 13% |

Reduction in still missed children, not available children, refusal, persistently missed children, and zero dose children in the current campaign round as compared to previous round

Increase in still missed children, not available children, refusal, persistently missed children, and zero dose children in the current campaign round as compared to previous round

Supplementary table 2. Area level impact of health camps in high-risk union councils of Karachi in August 2021.

Reduction in still missed children, not available children, refusal, persistently missed children, and zero dose children in the current campaign round as compared to previous round

Increase in still missed children, not available children, refusal, persistently missed children, and zero dose children in the current campaign round as compared to previous round

Supplementary table 3. Area level impact of health camps in high-risk union councils of Karachi in October 2021.

Reduction in still missed children, not available children, refusal, persistently missed children, and zero dose children in the current campaign round as compared to previous round

Increase in still missed children, not available children, refusal, persistently missed children, and zero dose children in the current campaign round as compared to previous round

**Supplementary Figure 1**

Supplementary Figure 1. The Union Councils included in each round of health camps in Karachi in 2021.


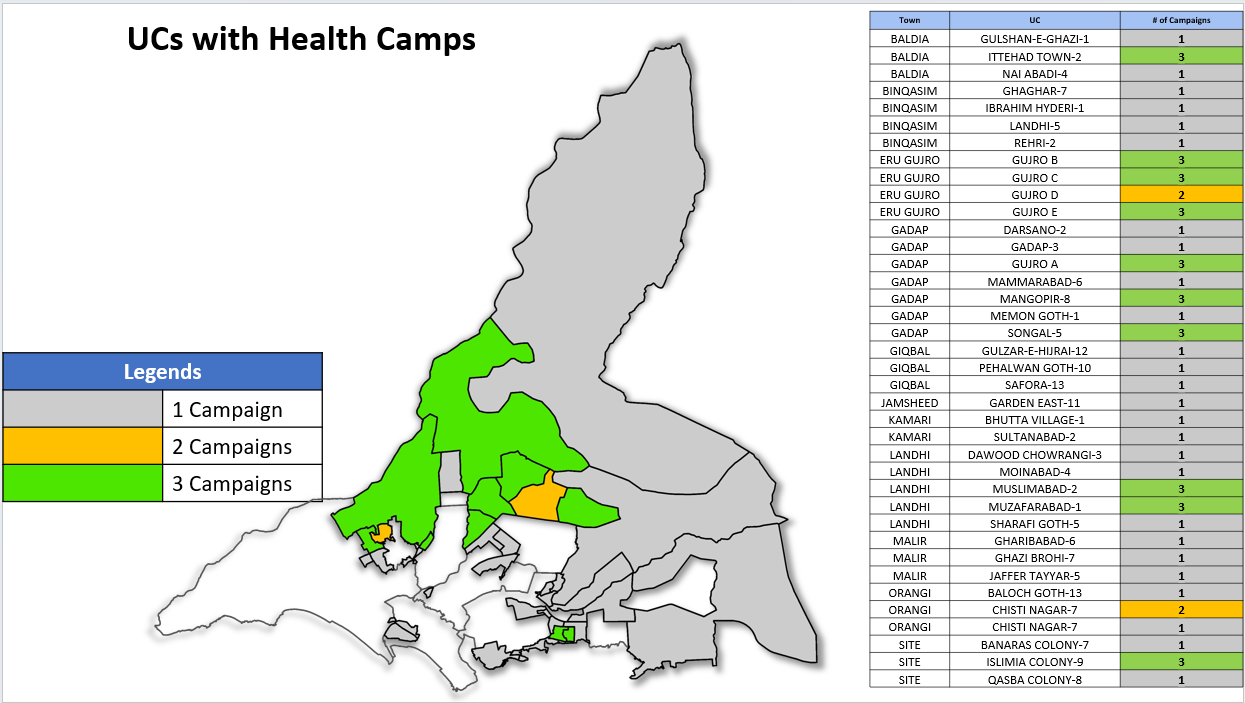

Supplement: Supplementary file 2 [file Data_Sheet_2.docx]
